# Identifying and Responding to the Complex Needs of Domestic Violence Housing Practitioners at the Onset of the COVID-19 Pandemic

**DOI:** 10.1007/s10896-020-00231-8

**Published:** 2021-01-05

**Authors:** Nkiru Nnawulezi, Margaret Hacskaylo

**Affiliations:** 1 University of Maryland, Baltimore County, MD USA; 2 National Alliance for Safe Housing, Inc (NASH), 712 H St, NE, Washington, DC, USA

**Keywords:** COVID-19, Intimate partner violence, Housing, Advocacy, Domestic violence

## Abstract

The social, financial, and emotional repercussions of the COVID-19 pandemic has left many organizations that support survivors of intimate partner violence questioning how to maintain core services while addressing compounding individual, organizational, and public health issues. Stay-at-home orders and other COVID-19 mitigation strategies have resulted in reduced shelter availability and increased intimate partner violence rates. Coupled with the economic impact of the pandemic, these factors have threatened financial and housing stability. To better understand these challenges and provide immediate support, The National Alliance for Safe Housing (NASH) co-hosted a peer support call to provide a virtual platform for practitioners to ask questions, discuss challenges, and share strategies for quality service provision during the COVID-19 pandemic. Over 800 practitioners from across the United States participated in the NASH call, most of whom were advocates, program directors, and managers. NASH gathered data on practitioners’ needs from a brief survey from the registration form analyzed using conventional inductive content analysis. Practitioners’ primary concerns were situated within eight questions, which we categorized into four meta-categories: (1) managing residential housing programs; (2) getting survivors materials resources; (3) keeping staff safe; and (4) maintaining organizational operations. The paper concludes with community-grounded and empirically supported practice recommendations aligned with practitioners’ expressed needs.

The rapid spread of SARS-CoV-2 (Severe Acute Respiratory Syndrome Coronavirus 2), the etiological agent of the illness known as COVID-19, is one of the most significant public health issues in the twenty-first century (Hartley and Perencevich 2020). Stay-at-home orders and social distancing requirements developed by state and city government officials promote public safety and prevent virus spread by reducing contact among residents. Stay-at-home orders mandate that people across the United States only leave their homes to complete essential activities, such as going to work, receiving crucial medical care, or shopping for food. Social distancing require that citizens stay at least 6 ft away from another person in outdoor and indoor spaces. At the onset of the implementation of these public health mandates, domestic violence practitioners, scholars, and activists expressed concern that stay-at-home policies would further exacerbate harm for survivors, or those living with intimate partners who sought to maintain power and control through the perpetration of violent tactics (Sharma and Borah 2020). Stay-at-home policies requiring survivors to remain confined in a home with their partners increase the risk of multiple types of abuse. Home confinement also compromises survivors’ ability to obtain help from supportive informal social networks, such as family and friends, or formal organizations, such as domestic violence shelters (Hall and Tucker 2020). The policies created to keep the general population safe also exacerbate harm in certain groups, spurring numerous practitioners and researchers to name this interaction between public health mandates and risk for increased violence as a safety paradox (Bradbury-Jones and Isham 2020). Our goals in this paper are to identity the specific needs of domestic violence practitioners negotiating this safety paradox, increasing the understanding of these needs, as well as providing recommendations to help maintain responsive, survivor-centered systems for sheltered and community-based survivor populations.

## Intimate Partner Violence in the Time of COVID-19

Intimate partner violence (IPV), also known as domestic violence, is comprised of certain behaviors exhibited by those individuals in a social hierarchy that seek to maintain power and coercive control over others with less power resulting from their gender identity (Wirtz et al. 2020). Groups with less power usually include women (trans and cis), femmes, and non-binary people. Given the forced confinement of partners as a result of COVID-19 related control measures, practitioners and scholars have identified IPV as an area of great concern during the COVID-19 pandemic. Practitioners who work with survivors have described that abusers were reportedly leveraging the power of stay-at-home mandates to expand their abuse of survivors (Hall and Tucker 2020). For example, to extend their control over survivors, some abusers were intentionally providing misguiding information about the parameters of stay-at-home orders or using fear-based tactics to exaggerate the risk of virus contraction leading to survivors’ further isolation and dependency on the abusive partner (Usher et al. 2020).

The confluence of stay-at-home orders and vigilant abusers has left survivors with few options to support their safety. Survivors generally use informal supports, such as friends and family, and formal supports, most commonly police officers, to cope with abuse and remain safe (Cho et al. 2020). Survivors usually call the police when they require immediate de-escalation support during an episode of severe physical violence (Nnawulezi and Murphy 2019; Dichter and Gelles 2012). While anecdotal evidence and news outlets across the globe have reported an increase in police calls for assistance with domestic violence, family disturbances, or family disputes during the COVID-19 pandemic, as of July 2020, only one study had systematically examined domestic violence-related police service calls. Using the Police Data Initiative database, cell phone location information, and restaurant reservation data across 15 major cities and counties in the United States, Leslie and Wilson (2020) found a 10.2% significant increase in domestic violence calls in the first two months after the institution of stay-at-home orders.

Systems formally designed to support domestic violence survivors also experienced swift and significant social, financial, and institutional repercussions as a result of the pandemic. Under normal conditions, community-based institutional responses include hotlines, personal protection orders, crisis shelters, and emergency housing support (e.g., hotel subsidies). Bed space in domestic violence shelters are insufficient relative to the need, but during the COVID-19 pandemic, shelter responses were exponentially more challenging to maintain given increased demand (Emezue 2020). Public health mandates also have complicated housing provision for survivors because they require crisis housing shelters to abide by and enforce social distancing rules among residents, quickly and significantly altering shelter intake policies. Many communal shelters were expected to find alternative placements for their residents to reduce the likelihood of viral transmission.

Finding alternative housing options proved difficult for many domestic violence organizations during the ongoing COVID-19 pandemic. In communications with technical assistance providers, shelter-based practitioners described how homeless shelter programs were not viable options because they struggled with the same placement issues. Some domestic violence shelter providers tried to use hotels as alternative lodging for survivors with mixed success. In many areas, hotels were being reserved for hospital overflow or to support primarily homeless individuals and families, leaving few available options for survivors.

At the onset of the COVID-19 pandemic, technical assistance providers received calls from domestic violence programs seeking guidance on how to quickly strategize ways to ensure the safety and health of their staff and survivors in their housing programs. Domestic violence programs had started following social distancing guidelines by operating with skeleton crews and implementing new cleaning protocols to prevent SARS-CoV-2 transmission, such as conducting frequent deep cleaning of their facilities. However, many programs did not have enough personal protective equipment to provide services safely while simultaneously facing budget reductions. Providers also asked numerous questions about changing shelter intake policies, responding to increased domestic violence calls, and coping with the rapid resource losses among organizations.

## Current Study

The pandemic’s quick onset led to crisis-orientated, reactive responses, making it clear that it was necessary to understand how to best support practitioners during the pandemic and subsequently improve program responses to survivors. This study explored the needs of practitioners who served IPV survivors during the ongoing COVID-19 crisis. While there has been a wide variety of editorials describing the nature of the problem, few empirical studies have identified the challenges with negotiating the safety paradox and responding to survivors’ immediate housing needs. In addition, no study to date has systematically documented the immediate impact of COVID-19 on program operations and service provision. This study seeks to fill this gap and provide practical recommendations on responding to the pandemic’s short and long-term effects on the domestic violence field.

## Methods

The National Alliance for Safe Housing (NASH), a national domestic violence and housing training and technical assistance organization, co-hosted an online meeting called “COVID-19 Resources and Response” in collaboration with its partners from the federal Domestic Violence Housing Technical Assistance Consortium. This meeting’s goal was to provide peer support and technical guidance for practitioners in the domestic violence field.

### Sample

NASH registered 840 participants for the national peer support call, and of those, 792 people identified their role in the domestic violence field. Thirty-five percent of respondents (*n* = 300) were executive or program directors. Almost 20% of callers provided direct service to survivors and identified as victim advocates or case managers (*n* = 162; 19.3%). Another 20% were program managers or coordinators (*n* = 172; 20.5%). The remaining 25% of callers represented smaller groups such as: technical assistance providers and grant administrators (*n* = 50); mental health professionals (*n* = 30); specialists (*n* = 28); supervisors (*n* = 17); attorneys (*n* = 14); presidents, vice presidents, and chief-level positions (n = 14),; and staff administrators (*n* = 12). Almost 6% (*n* = 48) of callers did not identify their position within the survey.

### Procedures

On March 13, 2020, NASH announced a peer support call about IPV during the COVID-19 pandemic that was sent to more than 1000 people through NASH and its partners’ listservs. The call was held on March 19, 2020, using the Zoom online meeting platform. Before the call, NASH sent each person a link to a brief survey. NASH downloaded these data from Google Forms into a Microsoft Excel file and sent de-identified data to the first author. The de-identified data was uploaded to NVivo 12 qualitative data analysis software for analysis. This study did not meet the institutional definition of research and was considered exempt from review by the university institutional review board.

### Measures

During the online registration process, NASH provided callers with the following prompt: “How can this peer support call be most helpful to you?” Eight hundred and three participants provided substantive responses to the question prompt. Incomplete responses, responses where the participant wrote “no answer” or “not applicable,” or responses that were one word (e.g., “yes” or “no”) were not considered substantive. About 89 participants provided additional responses beyond the initial prompt. Participants also provided their demographic information such as name, position, and their organizational affiliation.

### Data Analysis

We analyzed the participants’ responses about the pandemic’s impact on survivors and their programs using a conventional content analysis, a qualitative data analytic procedure that involves inductively coding text-based data to describe a phenomenon (Hsieh and Shannon 2005; Elo and Kyngäs 2008). To prepare for analysis, the first author familiarized herself with the data and maintained an audit trail throughout the analytic process to document emerging data patterns. The second step in data analysis was to complete open inductive coding using individual responses as the analysis unit. Once these initial codes were developed, the first author placed similar codes under meaningful higher-order categories with associated definitions, which would later become themes. Manifest content under each group of higher-order categories was checked to determine whether it aligned with the suggested category and associated meaning. Codes that were not thematically aligned were further refined or reassigned to a more relevant theme.. If there was alignment among the theme, code, and content, it was introduced to the second author to determine whether the emergent themes aligned with the field’s broader context. To establish the data’s credibility (as suggested by Hsieh and Shannon 2005), a thick description of the themes was presented to the second author, an expert in domestic violence.

### Reflexivity

The first author is a Black woman and a participatory research scholar with more than a decade of experience exploring how domestic violence organizations, specifically housing organizations across the domestic violence continuum, promote safety and power for survivors with multiply marginalized identities. The second author has more than 20 years of experience in creating, implementing, and managing programs for domestic violence survivors. She has specific expertise in developing a constellation of safe housing options for survivors that expand beyond traditional shelters. Both authors have a shared commitment to innovation and equity in practices, as well as to survivor self-determination, which influences how they shape future possibilities for the field.

## Results

Eight major themes arose from the data, and each theme is a question synthesized from collective responses from the survey. These questions are situated within a collective recognition of four essential needs, which are conceptualized as meta-categories: (a) managing residential housing programs; (b) getting survivors material resources (jobs, money, food, housing); (c) keeping staff safe; and (d) maintaining organizational operations.

### Category 1: Managing Residential Housing Programs

#### Question 1: How Can we Keep Survivors Who Are Living in Shelters Healthy?

More than a third of respondents wanted to know how they could maintain safety for survivors living in shelters when social distancing and quarantine requirements (e.g., keeping six feet apart, being in separate bedrooms, wearing gloves and masks) were not possible in communal residential spaces. Survivors often shared living areas, bathrooms, and sometimes bedrooms. Therefore, many respondents asked about ways to mitigate the high risk of virus transmission. Respondents demonstrated these ideas in questions such as: “What are best practices for housing programs and advocates to serve clients during the COVID-19 pandemic while also prioritizing staff health?” Respondents desired strategies to maintain safety while also having limited access to sanitation and other basic supplies. For example, a respondent stated: “[Can you tell us] how to access basic living supplies such as toilet paper being at the top [of the list] since our shelves and supply chains are out?”

In some cases, survivors continued to move in and out of the shelter because they were essential workers or had appointments in the community. Respondents wanted to learn tactics for supporting survivors in maintaining daily life activities while mitigating virus exposure risk for themselves and other survivors living in the shelter. A respondent wondered: “If [survivors] have traveled outside of our area to an area that has been hit harder with a lot more positive cases, what are the best procedures we should put into place when they are returning to our program and into their dwelling? For instance, [they have] interactions with their case managers, peer support from our team, and interaction with other participants.”

Shelter admission screening and intake procedures were other sources of confusion. Many respondents did not know whether they should continue to screen new residents, and if so, what questions they could ask before the person came into the shelter. One respondent said: “We have a centralized process for intake in our community. Some shelters are asking that we ask questions about if people have traveled or are feeling sick before we consider placing them in shelter. Is this a practice we should put into place?” Another respondent asked: “What do we tell participants who are showing cold/flu symptoms but [need] emergency services?” Other respondents had decided to alter their intake process but wanted advice on informing COVID-19-diagnosed survivors that their diagnosis would prevent shelter admission. Respondents also questioned whether they should screen shelter residents for COVID-19 symptoms and were primarily interested in the legalities and complexities of conducting health screenings with survivors. Many respondents simply desired to know how to support survivors who tested positive for COVID-19.

#### Question 2: How Do we Maintain Survivor-Centered Practices during Times of Mandates and Public Health Reporting?

Multiple respondents needed support to manage the discordance between encouraging survivor autonomy and establishing and upholding community health standards while operating shelters. For example, respondents desired guidance on the appropriateness of requiring survivors to wear a mask, get COVID-19 testing, or limiting their public movements. Some wanted to know what they should do if survivors refused to get tested or engage in the health care system.

Confidentiality is a critical component of survivor-centered care. Many respondents felt challenged by aligning with public health mandates to report COVID-19 cases while maintaining survivor and shelter confidentiality. Respondents were concerned about exchanging confidential information with referring community-based agencies. These sentiments were conveyed in one respondent’s multi-layered question: “What is the guidance around what a domestic violence shelter needs to do when (if) a single shelter resident tests positive for COVID-19? What are the expectations regarding alerting and testing other residents or participants who may have [contacted] the infected person? How does this potential need to share private and sensitive information get handled in light of confidentiality expectations and laws? Do we have to share with state/federal CDCs or public health organizations? If so, what information is shared and by whom?”

Some respondents also wanted strategies to ensure confidentiality while providing survivor advocacy over the phone or online services. For example, a respondent asked: “How can we ensure privacy, confidentiality with phone support for survivors of SA [sexual assault] while they are receiving care at an ER?” Another respondent stated: “Confidential communication – [how do we] limit face to face advocacy and look at providing assistance through technology or via phone [and] what happens when their partner or children are present, and they don’t have a safe place for that call?”

### Category 2: Providing Material Resources for Survivors

#### Question 3: How Do we Respond to the Significant Resource Loss that Survivors Are Experiencing, Specifically Housing, Jobs, and Money?

Survivors’ access to essential resources such as food, transportation, healthcare, housing, and employment have been threatened or eradicated because of the pandemic. Common requests from respondents were to identify or provide resources that could respond to survivors’ sudden losses. For example, one respondent asked: “What is the best practice in case a client gives us a call and [they] have a symptom with[out] medical insurance, a primary doctor, [or a] car?” Some wondered how the pandemic would impact survivors’ access to social services such as Supplemental Nutrition Assistance Program or Medicaid.

The most requested resource was housing support. Respondents wanted to identify short- and long-term housing for survivors unable to pay rent due to job loss or who were not admitted to a domestic violence shelter for exhibiting flu-like symptoms. Some respondents wondered how to help survivors if funding for other types of housing support, such as hotel voucher programs, were depleted. Respondents asked related questions such as: “With shelters closing and other means of housing diminishing (hotels), how do you help when options don’t exist?” or “What emergency housing alternatives are communities exploring with survivors outside of hotel/motel options?” There were also more complex requests regarding the distribution of housing resources, such as hotel rooms, given limitations in availability and health-related restrictions. Other respondents described how difficult it would be for survivors who experience sudden employment loss to maintain their place in housing programs that required strict rental contributions. A respondent wondered: “How has advocacy shifted in supporting survivors who have become permanently housed but are now faced with employment vulnerability? Are there homelessness prevention resources available to prevent reoccurrence?”

Some respondents asked for recommended resources to support survivors that would experience disparate COVID-19 impacts. For example, respondents identified populations who felt more vulnerable: survivors living on reservations, undocumented survivors, survivors with criminal histories, and non-English speaking survivors. Respondents desired to support survivors from specific communities with histories of multiple marginalization and heightened susceptibility to the pandemic’s harmful material, social, and health consequences. For example, respondents asked: “[How do we] address explicitly the impact of racial[ized] physical violence on East Asian survivors/communities in this COVID-19 crisis and its impact on access to services for sexual violence (SV) and IPV survivors?” and “How do national organizations plan on supporting smaller organizations, [such as] culturally specific DV organizations, who will have significant financial struggles during and long after this crisis is over?”

#### Question 4: How Can we Provide Effective Advocacy that Supports all Survivors, both Shelter-Based and Community-Based, to Stay Safe?

Many respondents sought to change the nature of their services from in-person to remote, especially advocacy services. Respondents were worried that the digital divide—the lack of equity in technological access—would impede many survivors’ ability to access resources or fully participate in services. Respondents asked questions: “How are we able to support survivors, not just via the phone, especially if we have a rural catchment area and a lot of our support is in-person?” and “How to host a support group for our survivors who rely on us when we aren’t offering in-person services?” Some had questions about filling out paperwork remotely or working with the elderly population who may have limited ability to navigate technology. Other respondents sought information about using social media to reach out to survivors who may need additional support.

Respondents wanted to know how to safety plan with community-based survivors, specifically those still at home with their abusers with no options for emergency housing and limited access to court services such as personal protection orders. For example, one respondent asked: “I would like to know how people are implementing safety plans when so many of the resources are limited or not available due to COVID-19 concerns.” Safety planning also requires reliance on other community supports, which were either temporarily closed or operating at a limited capacity. Given these limitations, respondents desired new and innovative ways to continue to engage in systems advocacy, as was evident in this respondent’s question: “Could you please provide examples of how service providers are creatively responding to ensure consistent critical support for survivors of domestic and sexual violence during this challenging time?” Innovations in providing critical support felt necessary for many respondents who were unable to provide in-person services, had a scarcity of internal organizational and external community resources, and had no idea about when these resources would return “back to normal.”

### Category 3: Keeping Staff Safe and Well

#### Question 5: How Do we Navigate Keeping ourselves Safe while Working with Survivors?

Respondents desired risk mitigation strategies that could keep both survivors and practitioners safe. This concern was one of the most prominent questions in the data. Many respondents were interested in knowing how to do their jobs effectively but also safely and with compassion. For example, one respondent asked: “How can we mitigate the virus risk (the elephant in the room) while being caring and helpful in the presence of a client?” This concern was especially valid for staff that continued to provide in-person life-saving advocacy and wanted to support survivors with the least amount of disruption but also desired to minimize exposure and risk to advocates. One responded described: “We still want survivors to obtain housing (especially because they will be safer housed than homeless), but how do we support advocates in protecting themselves while also supporting survivors to move into new housing?” Others asked about having to travel safely within the community to continue supporting survivors, especially practitioners who worked in hospitals. One respondent wondered: “How are ER visits for victims of IPV and SA being prioritized right now? What is the best way to advocate to ensure a client is receiving appropriate care while staying safe from potentially infected individuals?” Some respondents asked how they could best support an advocate who developed COVID-19 through their subsequent quarantine. A few respondents also wanted to know when advocates should be allowed to return to work and whether program staff should notify other staff that they contracted the virus.

#### Question 6: How Can we Support Survivors and Staff Who Experience Anxiety, Panic, and Other Adverse Emotional Reactions to COVID-19?

Respondents desired specific information and tools on how to support survivors and advocates with emotional responses such as anxiety and panic due to unstable and uncertain times. Specifically, a few respondents asked for ways to give information to survivors that would not traumatize them or encourage further panic: “What are the best practices associated with disseminating crisis information to clients that mitigate anxiety and further crisis for a client?” Respondents also described being anxious about their own health and the health of survivors. They desired ways to promote calm and resiliency and address the vicarious trauma that arose from serving survivors. One respondent stated: “What more can we do to support staff whose roles are causing them to be exposed but who are also personally experiencing a high level of anxiety towards being exposed?” Some described the need for more information about self-care strategies and opportunities to infuse hope for survivors and staff.

### Category 4: Maintaining Organizational Operations

#### Question 7: How Can we Create Supportive and Responsive Workplaces while Also Attending to the Realities of Program Operations?

Numerous respondents wanted to learn how to create a work environment that was safe, comfortable, and responsive to the current pandemic, while also appropriately attending to staffing and budget-related issues and workload expectations. Staffing issues arose when asking about shelter operations. Many respondents wondered how to handle labor shortages if staff quit their jobs or did not show up for work. Others worried about having to fire staff or cut hours due to budget limitations or potential shelter closures. Respondents asked questions specifically about how to keep the shelter running with limited staff. Many described not having enough staff willing to risk virus exposure to support efficient program operation. One respondent asked: “I am fielding questions about staff not feeling comfortable in the space, but the shelter has to stay open and must be staffed, even if that means staff availability looks lighter. How do we balance this?” Another respondent asked: “How can we admit new clients into a shelter —we have staff who are at high risk and we are putting them in an unknown situation—what happens if staff refuse to come to work as they believe their workplace is not safe?” Respondents struggled with handling staff who decided to take personal leave or, given the risk of working in close proximity to other people, decided to quit their jobs. Respondents also wondered how to deal with staff getting sick and being unable to cover their shifts.

In addition to staffing, respondents desired to know whether additional funding would be made available to shelters to address the financial burdens that arose as a result of the COVID-19 situation. Respondents needed funding for extra supplies, extra food, paid time off for staff, and infrastructure. Specifically, respondents wanted to learn about obtaining additional funding to cover hotel costs for programs that could not house survivors at the shelter. For example, one respondent stated: “Many of our clients are not getting shelter as new clients are not being admitted. In this situation, how far can we sustain in providing hotel assistance? The hotels will also need additional assistance like food, which the shelter usually takes care of. Will there be additional funds available to DV/SA agencies to address these issues?”

A fewer number of respondents wanted to know how to talk to funders about supporting flexible practices and specifically desired advice about convincing funders to waive requirements around allowable housing expenses, provide flexible food support, and pay for hotels for those not admitted into shelters. Some wondered how to support remote advocacy that was expected to be done in person: “What do we do if our grants don’t support work done remotely?” They wondered whether funders would continue to support teleworking staff and how to translate job activities into a format conducive to working from home.

Questions about workload were common among respondents. Many respondents wondered what job expectations were considered reasonable, given the shifting responsibilities due to the pandemic. Respondents asked: “What additional tasks are you providing to direct-service folks in order to ensure they can fill an eight-hour workday, and thus still be paid?” or “Our local programs have questions around providing administrative leave pay to advocates when they cannot work their full hours due to working remotely.” Other respondents had questions about providing hazard pay to shelter staff or non-essential workers becoming essential workers to make up for staffing loss. One respondent stated: “Since some staff are able to work from home, while shelter staff are asked to work on site, what are recommended pay increases for those staff who are taking on more risks to serve our survivors?”

Respondents were concerned about how to navigate the potential of closures of their organizations as well as other organizations within their ecosystems. Some respondents asked questions such as: “How can we, as services providers, be of any assistance to clients when many places are closed?” Other respondents desired to know about how to continue supporting survivors despite potential closures: “What happens if we become so low on staff that we have to shut down programs? We have staff that may have childcare issues, become sick, etc.”

#### Question 8: We Are our Greatest Resource. What Are some Ways we Can Support each Other and our Communities?

The respondents’ most commonly stated desire was to understand how other organizations were handling their COVID-19 responses. Many respondents sought to learn how other programs were delivering services or developing or adapting protocols. Respondents wanted to share tools and information that had been working for them. One respondent wrote: “I get to hear the perspectives from other advocates, form a bond with other advocates and other professionals in this field. We can all learn from each other and lean on one another. This field is a challenging field. We can support one another, become strengthened to help our clients.”

While some respondents were interested in learning how their colleagues were handling COVID-19-related challenges, others wanted information on how to be a resource to members in their immediate communities who were not in the movement but were supporting survivors. Respondents were particularly interested in staying informed about the specifics of COVID-19 pathology and disease dynamics to accurately share information with others. Respondents specifically were interested in its exact symptoms and lethality, illness duration, infection risk, recommended treatment approaches, and effective transmission control and mitigation strategies.

A few respondents wanted to ensure that they were communicating a unified message around the state of the domestic violence field. One respondent was particularly interested in finding the right messaging to share with the community: “How do we communicate with the public at large about how to support DV survivors if programs are closing their doors?” There were a few respondents who wanted to know how to talk about the impact of COVID-19 with survivors and community members. One respondent asked for specific language to enable transparency about the potential limitations of their provided services: “What can we expect from this situation? What can we tell clients/residents in terms of worst-case/best-case scenarios in order to be transparent about our capacities, limitations, and possible hurdles should things get worse, or when things can get better?”

Respondents who self-identified as technical assistance providers desired to support member programs and provide information about appropriate policies and practices to implement. Many technical assistance providers and funders were interested in understanding and responding to program needs. For example, one respondent stated: “As a funder, how should I prioritize support to my grantees that provide domestic violence services? What are the greatest needs?”

## Discussion

This article identified the needs of practitioners in the domestic violence field during the COVID-19 pandemic. As essential workers, practitioners were concerned about keeping themselves, their staff, and survivors healthy while providing housing services in conditions that were not well-suited for social distancing. They desired internal and external tools to assist their responses to the compounding effects of limited crisis housing, survivors’ material losses, and changes in staff, workload, and advocacy requirements. Many described having difficulties in determining how to continue advocacy that centered survivors’ diverse needs while also reducing IPV-related and pandemic-related trauma. Respondents were also aware that some survivor communities were disproportionately impacted due to inequitable and oppressive social systems and wanted to be responsive to these diverse needs. Overall, practitioners conveyed a palpable desire to be innovative and expansive in their practices.

Despite the study’s novelty and its responsiveness to the current sociopolitical context, there were some limitations. Rapid data collection did not allow for a second coder at the open coding stage. Usually, a second coder at that stage allows for greater coding accuracy, enhancing trustworthiness. However, the second author reviewed the coding structure and content during the categorization stage and was responsible for collecting the data and facilitating the peer review workshop.

### How to Move Forward: Implications for Practice, Policy, and Research

In pre-COVID-19 pandemic conditions, domestic violence programs were designed to support survivors in meeting their basic needs—food, water, shelter, material resources and goods, clothing, sleep, health, and safety, while working toward higher-order goals—long-term healing from trauma, improved education and incomes, and achieving personal empowerment and fulfillment. This approach aligns with fundamental motivation theories, which state that humans prioritize basic needs before other desires (Maslow 1943). These goals may make sense when there are limited threats to basic resources, community partnerships are more stable, and routines are more predictable. However, the pandemic has threatened or impeded survivors’ ability to achieve these desired outcomes, leaving practitioners with a renewed focus on centering survivors’ basic needs to ensure survivors remain safe and healthy. However, our results demonstrated the tensions inherent in focusing on survivors’ basic needs while simultaneously addressing their own needs. Based on study findings, we provide five recommendations that support survivors’ basic resource attainment while also responding to the need for innovations in practice.

### Recommendation 1: Provide Low-Barrier Access to Shelter Services with Individualized, Flexible Services

As the demand for domestic violence shelter services has increased while resources have decreased, programs need to have nimbler intake policies and rapidly triage service requests. Original rigid shelter rules were and will continue to be challenging to maintain during social distancing. Practitioners reported needing support in managing shelter policies and regulations and meeting survivors’ basic resource needs. Therefore, the first recommendation is to abandon the rigorous screening and eligibility routines and adopt a faster, low-barrier approach. A low-barrier approach encompasses a set of policies and practices that seek to remove service access barriers, providing a robust framework through which staff can expeditiously serve survivors living in the community (Nnawulezi et al. 2018). The focus on expedient service delivery from formal support systems aligns with recent recommendations from domestic violence researchers (Sharma and Borah 2020).

Low-barrier policies specifically reduce bureaucratic requirements for documentation at shelter entry to streamline intakes. This reduction is consistent with the National Network to End Domestic Violence guidelines for communal programs during the pandemic and includes dropping the requirement for survivors’ clean health screenings to receive services (National Network to End Domestic Violence 2020). Many providers can also reduce or eliminate time limits for shelter stays, as well as for standards for income, employment, or other program goals. Low-barrier policies attend to survivors’ need for greater flexibility, particularly during the pandemic, due to the lack of alternative housing options available to them at the end of their stay. Given the reality of limited housing, many state governments and other funders support these flexible, low-barrier measures. They are consistent with moratoria instituted through the Coronavirus Aid, Relief, and Economic Security (CARES) Act in the United States. In addition, employing techniques to determine eligibility and conduct intakes electronically will better enable programs to ensure staff safety. For example, some domestic violence programs have restructured their intake processes entirely to employ low-barrier, electronic decisions. Using strategies such as deferring signatures on paperwork that can either be completed electronically or after workplaces re-open minimalize processing time, producing powerful and immediate access for survivors of diverse backgrounds.

### Recommendation 2: Provide Flexible Financial Assistance and Support Mutual Aid Responses to Support Survivors’ Housing and Economic Stability

In this study, respondents described survivors who experienced significant pandemic- related economic consequences, such as recent unemployment, which affected their ability to pay rent, buy food, and access transportation. Survivors’ significant resource losses were interconnected to practitioners’ need to help survivors access basic resources. The rapid deterioration of social service, healthcare, housing, carceral, and economic systems within a few short weeks after the pandemic’s onset made apparent the need for policies that support universal attainment of essential resources such as basic income, housing, and healthcare. Investments in universal social supports, such as basic income or healthcare, are ways to mitigate the immediate repercussions of a healthcare crisis.

Until the provision of universal support, one of the field’s most promising interventions designed to mitigate housing and economic crises and maintain stability is flexible funding (Sullivan et al. 2019). Flexible funding comprises small, low-barrier grants given to survivors to help them quickly obtain or maintain independent housing. During the pandemic, many programs have been advocating for flexible funding assistance in the absence of adequate shelter bedspace and to meet essential needs for community-based survivors. For example, programs used flexible funding to assist survivors with COVID-19-related needs, such as health care expenses, medical and cleaning supplies, and personal protective equipment such as masks and gloves. This quick, flexible, and targeted assistance enabled programs to help survivors remain safe and stable in their communities, while also allowing programs to maintain contact with at-risk survivors during the crisis. For survivors who lost their jobs or income support due to the disruption in public services, flexible funding was often the only way to ensure their safety and ability to sustain their families, especially for undocumented survivors and those unable to access mainstream support sources.

Mutual aid societies are another innovative way for survivors to obtain their essential needs within their communities (Dominguez et al. 2020). They are particularly useful for those isolated in their homes with their abusers. While programs strived to meet increased service demands, it was impossible to engage with all survivors in the face of reduced staffing and resources. However, mutual aid efforts provide community members with vital supports through existing community networks. Domestic violence organizations can provide virtual training to mutual aid volunteers to engage in safety planning and check-ins with community members. When adequately resourced and administered without caps, restrictions, and other limitations, flexible funding and mutual aid responses are exceptional strategies for maintaining survivor-centered practices while also responding to their significant resource loss, both during and after the pandemic.

### Recommendation 3: Offer Dynamic, Comprehensive, and Survivor-Led Safety Planning that Supports Choice

Safety planning that is ongoing, responsive, and comprehensive best aligns with the complexity of survivors’ lives and increases their access to resources. Amid the pandemic, safety planning had to include working with survivors to develop contingency plans and consider caretaking responsibilities in case of a COVID-19 diagnosis. Advocates can help survivors connect with community programs to plan for how to shelter-in-place safely or relocate to alternative housing.

Similar to dynamic safety planning, programs that enable survivors to choose which supports they need, instead of offering a one-size-fits-all approach, often provide more expedient responses to survivors (Kulkarni 2019; Stylianou 2019). As COVID-19 persists, many providers are assisting survivors beyond the need for safety and housing, including support for children’s services and homeschooling, as well as employment and income support. Advocates seek to remain connected to survivors in the community through food, medical, and health supplies deliveries, rather than only virtual contact. Holistic, ongoing safety planning and relying on survivors’ right to choose what they want for their lives are values integral to survivor-centered services and necessary to continue to integrate during COVID-19 and beyond.

In addition to supporting survivors by increasing access to support and resources, flexible mobile advocacy also helps keep staff safe. Mobile advocacy, or the provision of advocacy services that rely on advocates meeting survivors in the community, is a relevant and empirically supported strategy compliant with government mandates to limit virus exposure without requiring survivors to meet in offices (Sullivan and Olsen 2016). Instead of strict policies and procedures, many programs increased their online and phone communication with survivors and staff to ensure clear and consistent contact. This communication may include the use of smartphone applications (Augusto de Lima et al. 2020) or virtual and text-based advocacy support (Viveiros and Bonomi 2020). However, many providers must be mindful that abusive partners may also use technology to harm survivors or increase survivor surveillance (Emezue 2020). Suggesting increased technology use to survivors should consider its potential benefits and disadvantages, specifically its potential iatrogenic effects on survivors’ safety.

### Recommendation 4: Provide Safe Housing Options through a Range of Models and Settings

Providing survivors with housing services outside a shelter, as many programs are being forced to do, supports survivors’ long-term housing stability and safety. In this study, multiple practitioners expressed concern about responding to survivors’ critical and immediate housing needs. As shelter programs work to accommodate increased privacy needs amid social distancing and other public health recommendations, many quickly needed to match appropriate housing options with survivors’ needs. Program staff had to identify hotels and other short-term lodging options that would accept survivors for limited stays. However, it was difficult for program staff to determine the most appropriate length of stay during the pandemic, resulting in many programs eschewing limits on lengths of stay, depending on their funders’ and the lodgings’ flexibility. Other non-conventional methods of safely housing survivors include developing partnerships with homelessness sector housing providers to facilitate entry into these programs when domestic violence-specific housing becomes inaccessible. In addition, organizations can provide free legal support to survivors to remain in their own homes and access legal protections to remove abusers (Breckenridge et al. 2016). These strategies have been used for years in areas where shelter programs are not readily available.

The growing problem of limited housing availability has resulted in more programs quickly learning how to advocate for general housing protections afforded under the CARES Act, including helping survivors access rent and mortgage moratoria, obtain waivers of public housing requirements, and extend housing voucher search time limits. Providers also increased outreach to private landlords in their communities to help broker lease-ups for survivors in affordable housing.

Culturally specific programs have also historically worked to become integrated with community-based housing efforts to attend to survivors’ unique cultural contexts and develop options that allow them to divest in carceral responses to violence. Such organizations and programs have recognized how the criminal and legal systems perpetuate housing instability and exacerbate harm for survivors. Therefore, they have developed and advocated for alternative incarceration responses, such as whole-family housing, lease bifurcations when violence occurs, and alternative housing options for people who cause harm. There is also advocacy to eliminate policies such as nuisance ordinances that lead to survivor displacement or changing program definitions of “fleeing” which disproportionately de-stabilize survivors’ families. Moving toward a constellation of safe housing options that consider survivors’ specific family configurations and social supports is an innovative strategy that should be fully adopted into the IPV field.

### Recommendation 5: Develop a Trauma-Informed Organizational Culture that Promotes Autonomy

Embracing trauma-informed care organizational principles and practices will support practitioners’ immediate need to maintain operations while also ensuring staff health. Designing a responsive and supportive workplace means creating a flexible environment where individuals are respected, have agency over their roles, and are routinely acknowledged for their strengths. Organizations should focus on enabling staff to understand the dynamics and nature of trauma and emphasizing connections among members of the organization in order to accomplish their goals (Wilson et al. 2015). Leadership can increase staff agency, provide flexible work routines, and expand possibilities for virtual advocacy, which can enable increased flexibility with survivors.

Many programs can employ online staff gatherings and peer support calls for staff to share their pandemic-related concerns, questions, and management ideas during the pandemic. Some programs can also increase their communications between managers and advocates to ensure that all providers receive ongoing support and do not become isolated during their new remote work routines. To maintain shelter operations while being mindful of increased cognitive, emotional, and physical workload burdens on staff, some programs could hire contractors for specific functions performed by staff previously, such as property management and food delivery. Trauma-informed cultural and structural adaptations directly attend to staff concerns about keeping themselves safe from infection, balancing work and family needs, and managing the stress of providing essential services during the pandemic.

Some scholars have also discussed the need to continue to provide resources to the social service systems and the individuals who keep them going by providing them personal protective equipment and ensuring full pay during the pandemic or providing additional hazard pay and paid time off (Bradbury-Jones and Isham 2020). Many shelter programs have worked diligently to respond to survivors’ needs within the rapidly changing times, stoking providers’ expressed concerns about whether funders would support the shifts away from conventional shelter housing approaches. Government funders at the county, state, and federal levels should follow the rapid innovation of the field and support implementing these innovations and ensure the systematic documentation of the strategies that arise from this era. Explicit commitments to fund these innovations in practice would reassure programs that their concerns are being heard. Private philanthropy can also demonstrate their support through robust and multi-year funding portfolios to ensure that these changes may be embraced for the long-term.

## Conclusion

COVID-19 has pushed practitioners to figure out methods to increase survivors’ access to support and safety, despite limited resources and health-related restrictions. Many domestic violence programs have had to innovate – in real-time and on the ground, without the benefit of advance planning or additional resources and with a rapid decrease in the availability of crisis housing services. Therefore, practice innovations in housing access require a full integration and continuous adoption of practices that center flexibility, freedom, housing, money, choice, and compassion. Creating increased access methods in response to the pandemic and maintaining these innovations post-pandemic will enable more survivors to receive basic resource support, especially communities of survivors who experience multiple marginalization.

This article aims to call practitioners and researchers in the domestic violence field to systematically document, identify, and evaluate the creative and innovative strategies that programs, practitioners, and survivors are currently using to navigate issues and maintain housing. It is essential to identify, target, and bring to scale community-based housing strategies that can withstand social, cultural, and political shifts. These strategies support the field’s collective goal to cultivate a world where all survivors are well-resourced, and IPV is eradicated.
